# 950. The Challenge of Fellowship Interviews During COVID-19: An Online Post-interview Survey

**DOI:** 10.1093/ofid/ofab466.1145

**Published:** 2021-12-04

**Authors:** Rattanaporn Mahatanan, Maxwell Vergo, Richard A Zuckerman

**Affiliations:** 1 Dartmouth Hitchcock Medical Center, Lebanon, New Hampshire; 2 Dartmouth-Hitchcock Medical Center, Lebanon, New Hampshire

## Abstract

**Background:**

COVID-19 has significantly disrupted social and personal interactions, including fellowship recruitment. In-person interviews were replaced by virtual interviews, which created uncertainties for both programs and candidates. We distributed a survey to interviewees for fellowship programs in an effort to gather data and improve the process.

**Methods:**

An anonymous online survey on the Qualtrics**®** platform assessed satisfaction with the interview process, evaluated the advantages and disadvantages of virtual interviews, and requested comments to improve the process. Surveys were sent out to candidates within 7 days of interview for Infectious Disease and Palliative care fellowship programs at our institution.

**Results:**

Surveys were sent to 51 candidates, 24 (47%) responded; 8 (33%) from Palliative care and 16 (67%) from ID. All candidates felt that they had a good sense about the programs and enough information to make a decision for ranking. Most candidates felt that they conveyed themselves well (71%) to very well (25%) during interview except one person who did not. 63% of candidates felt that the process was seamless, although 3 (12.5%) mentioned technical difficulties during the interviews. While 79% felt that the time spent on the interview was about right, 16% of candidates felt that interviews were too long and 1 person felt that it was too short. Cost-saving was the top advantage of the virtual interview with time-saving second. Interestingly, ability to interview at more programs was not ranked as highly as an advantage. Despite these advantages, 19 of 24 (79%) of candidates would have preferred an in-person interview if it was available. Lack of personal interaction and inability to see the location were equally chosen to be the greatest disadvantages of the virtual process by most of the candidates.

**Conclusion:**

Despite the challenges of the virtual interview process, our survey showed positive feedback from candidates regarding their experiences. The lack of social interaction and inability to explore the location were important, prompting 79% of candidates to prefer an in-person interview if that was an option. Many factors should be considered to ensure an equitable and comprehensive process where candidates and programs can make decisions to optimize outcomes.

**Disclosures:**

**All Authors**: No reported disclosures

